# Self-motion perception and sequential decision-making: where are we heading?

**DOI:** 10.1098/rstb.2022.0333

**Published:** 2023-09-25

**Authors:** Steven J. Jerjian, Devin R. Harsch, Christopher R. Fetsch

**Affiliations:** ^1^ Solomon H. Snyder Department of Neuroscience, Zanvyl Krieger Mind/Brain Institute, Johns Hopkins University, Baltimore, MD 21218, USA; ^2^ Center for Neuroscience and Department of Neurobiology, University of Pittsburgh, Pittsburgh, PA 15213, USA

**Keywords:** vestibular, visual, self-motion, navigation

## Abstract

To navigate and guide adaptive behaviour in a dynamic environment, animals must accurately estimate their own motion relative to the external world. This is a fundamentally multisensory process involving integration of visual, vestibular and kinesthetic inputs. Ideal observer models, paired with careful neurophysiological investigation, helped to reveal how visual and vestibular signals are combined to support perception of linear self-motion direction, or heading. Recent work has extended these findings by emphasizing the dimension of time, both with regard to stimulus dynamics and the trade-off between speed and accuracy. Both time and certainty—i.e. the degree of confidence in a multisensory decision—are essential to the ecological goals of the system: terminating a decision process is necessary for timely action, and predicting one's accuracy is critical for making multiple decisions in a sequence, as in navigation. Here, we summarize a leading model for multisensory decision-making, then show how the model can be extended to study confidence in heading discrimination. Lastly, we preview ongoing efforts to bridge self-motion perception and navigation *per se*, including closed-loop virtual reality and active self-motion. The design of unconstrained, ethologically inspired tasks, accompanied by large-scale neural recordings, raise promise for a deeper understanding of spatial perception and decision-making in the behaving animal.

This article is part of the theme issue ‘Decision and control processes in multisensory perception’.

## 1. Introduction

Consider the challenge of scaling a wall at your local rock-climbing centre. A successful, fast, climb to the top is facilitated by estimating an optimal route from an initial vantage point (or several) on the mat. During each movement across or up the wall, multiple sensory inputs are available to the brain to guide a successful climb: vestibular signals arising from motion of the head through space; visual signals from motion of the scene across the retina; proprioceptive and tactile signals indicating the position and motion of the limbs and the quality of a hand- or foothold. Small or slippery holds may render tactile information unreliable. Visual input could be ambiguous or uncertain, for example if one is climbing on an overhang or with reduced ambient light levels. Depending on the frequency and amplitude of head motion, vestibular inputs may be unreliable or fail to disambiguate translation from tilt. Thus, to estimate their ongoing motion with respect to the goal and select actions accordingly, the optimal climber will use information from all available sources, at each moment instinctively leaning more heavily on the more reliable ones.

**Figure 1.**
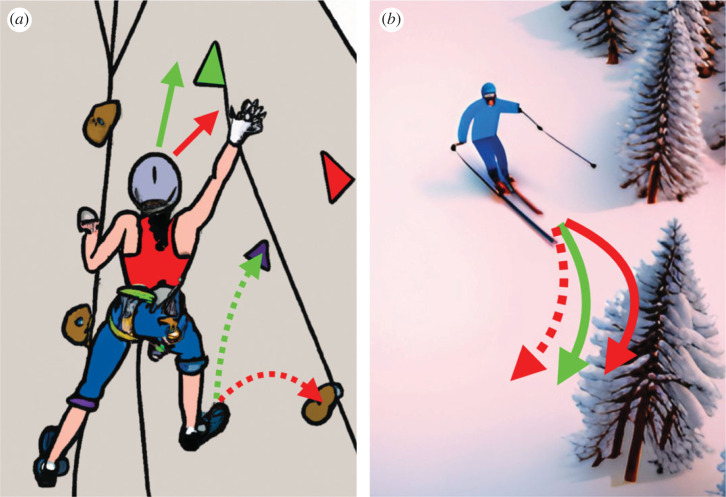
Sequential self-motion decisions and the role of confidence. The brain executes a motor plan to reposition the body along a desired trajectory. Multisensory feedback allows perceptual judgement of the actual trajectory, but this may not be exactly as planned. (*a*) For the climber, the intended trajectory (green solid arrow) affords grasping the green handhold and advancing the foot upwards (green dashed arrow), but if the trajectory turns out to be more lateral (red solid arrow) this could prompt a more conservative approach (red handhold and dashed arrow). A low degree of confidence in the initial heading judgement would recommend the conservative strategy. (*b*) Similarly, the skier intends to direct her turn just in front of the next tree (green), but in actuality might be heading dangerously close to it (solid red). Low confidence should cause her to hedge and keep a more comfortable distance (dashed red). High confidence followed by a negative outcome (a branch to the face) should trigger adjustment of an internal model or sensory-motor mapping. Illustrations generated with the help of AI tools (DALL-E and Jasper AI).

With each self-motion judgement, two other features of a perceptual decision are at play. First, the timing of commitment to a decision about one's direction of motion must itself be decided. Fast decisions during a climb could yield a quicker finish, thereby conserving energy or winning a competition—but committing too early during movement risks a dangerous error. Second, a climber's confidence that they have made an accurate self-motion judgement is also critical. Low confidence may lead to a more tentative reach, allow for a re-evaluation or adjustment of trajectory (figure 1), or leave open the possibility of reverting to a previous position. Higher confidence, on the other hand, could drive a quicker motion and firmer limb placement, or allow for a riskier manoeuvre with a greater pay-off in terms of positioning for ultimate success. In a reinforcement learning framework, confidence can be seen as a critical modulator of learning rate, or as impetus for revising an agent's internal model [1,2]—for instance if a high-confidence decision is revealed to be an error, it means something about the world has probably changed.

In the following sections, we first summarize existing work on the integration of visual and vestibular cues for heading perception and discuss the importance of considering decision speed and confidence in multisensory decision-making. We present preliminary findings in a heading discrimination task that combines measurement of choice, reaction time (RT) and confidence, which may be considered a bridge towards more complex tasks with multiple decisions forming a hierarchical sequence. We then review the recent development of naturalistic paradigms which can be used to study self-motion perception during target-tracking and virtual-reality (VR) navigation. When paired with continuous behavioural monitoring and multi-area neural recordings, these ecologically inspired paradigms promise unprecedented neurobiological insights into spatial cognition and guidance of movement in the real world.

## 2. Visual-vestibular integration: *raison d'être* and neural substrates

Although vestibular sensations rarely impinge on conscious awareness under healthy conditions, the system continuously signals linear acceleration of the head in space [3] via the two otolith organs in each inner ear. Owing to fundamental physical constraints captured by Einstein's equivalence principle, the otoliths alone cannot distinguish translational inertial motion from a change in orientation relative to gravity. This ambiguity can be resolved using signals from the semicircular canals, which detect angular acceleration in three orthogonal planes [4]—although canal afferents themselves are relatively insensitive to low-frequency motion and static tilts [5]. These physical limitations can give rise to phenomena like the somatogravic illusion, the erroneous perception of linear acceleration as tilt [6]. Separately, scene motion across the retina (optic flow) can cause a profound sensation of self-motion, and has long been studied as the visual basis of heading judgements [7,8]. Yet vision also has shortcomings: the information quality of optic flow can vary dramatically with viewing conditions or behavioural context, and the visual system must contend with confounding movements of one's own eyes and head, and moving objects in the environment [7,9].

Fortunately, under most real-world conditions, informative signals are available from both vestibular and visual senses. If these signals arise from a common source, such as intentional self-generated locomotion, the information they provide is generally convergent. Since each source is encumbered with limitations and ambiguities it cannot resolve alone, it is natural to consider combining information from both when possible.

Indeed, monkeys and humans can accurately and precisely perceive heading from either visual [10] or vestibular cues [11], and performance improves when both are presented together [12,13], suggesting that these two sensory cues are combined to improve heading perception. In theory, statistically optimal integration (Bayesian or maximum-likelihood estimation (MLE)) [14–16] is realized by weighting the cues according to their reliability, and empirical investigations demonstrated behavioural signatures of near-optimal cue integration in a multisensory heading discrimination task [12,13]. This classic task has been used for a comparative understanding of human and non-human primate self-motion perception, and is well suited for the investigation of its neurophysiological correlates, through invasive recordings in trained macaque monkeys (see also Zeng *et al*. [17]).

A number of cortical areas with selectivity for visual and vestibular heading stimuli have been linked to a network for self-motion perception in both humans and monkeys [18,19]. Key nodes include the dorsal medial superior temporal area (MSTd), ventral intraparietal area (VIP), the smooth pursuit region of the frontal eye fields (FEFsem), and a multimodal region of the posterior sylvian fissure (VPS). Vestibular and visual motion signals, arriving from unimodal areas such as parieto-insular vestibular cortex and the middle temporal (MT) visual area, respectively, are thought to converge in these downstream multisensory areas [20,21]—although direct projections from MT to parts of the network other than MST and VIP have not been verified.

MSTd has been of long-standing interest in particular [22], as it contains a population of neurons with congruent selectivity for visual and vestibular heading. These neurons show striking correlates of both the increase in perceptual sensitivity during cue combination, and reliability-dependent cue weighting [12,23–25]. On the other hand, MSTd neurons lack a common spatial reference frame for visual and vestibular information (they remain eye- and head-centred, respectively) [26], although this may not be a necessity for effective integration [27]. Furthermore, although MSTd's velocity-dominated vestibular responses suggest a temporal integration from the periphery to match the dynamics of visual motion signals [28–30], reversible inactivation of MSTd has minimal effect on vestibular heading judgements [31], and downstream decision-related activity tracks vestibular acceleration [32]. Thus, while recordings in MSTd support some theoretical predictions regarding optimal cue combination, there remain inconsistencies and open questions regarding its role and the nature of network-level interactions subserving heading discrimination [33,34].

Other areas have their own quirks. The multimodal population in VPS is dominated by neurons with opposite tuning to the two modalities, implying a greater role in segregation than combination [35]. Area VIP, meanwhile, shows strong choice-correlated activity, but surprisingly no apparent causal role in heading discrimination [36]. To date, neurophysiological studies have shed some light on the possible functions of each area, but there remains work to reconcile various models of where and how near-optimal cue integration is achieved.

### (a) Limitations of the conventional definition of optimality

Following the lead of earlier research, many of these studies implicitly assumed that subjects use all the information available throughout the stimulus presentation—or at least that the same subset of the presentation epoch is used for the unisensory and multisensory conditions. This assumption matters because testing normative models of cue integration generally requires estimating the reliability of individual cues to generate predictions for the multisensory percept; namely, how the cues should be weighted, and how much more precise the multisensory estimate should be. However fixed-duration tasks permit the decision to be formed covertly at any time during stimulus presentation [37,38], and at different times for different trials/conditions, leaving it unclear how to compute the predictions for an optimal observer.

For instance, the classic Bayesian/MLE approach defines optimality as maximizing the precision of the combined estimate, given the precision of the unimodal estimates. For linear weighted integration with uncorrelated inputs, the MLE prediction is captured by the equation: $\sigma_{c}^{2}=(\sigma_{a}^{2}\sigma_{b}^{2}/(\sigma_{a}^{2}+\sigma_{b}^{2}))$, where $\sigma_{c}^{2}$ is the variance of the combined estimate, and $\sigma_{a}^{2}$ and $\sigma_{b}^{2}$ are the variances of the unimodal estimates. Typically, performance on unimodal conditions is used to estimate the reliability of the signals being combined, and the prediction from the above equation is compared to performance in a multisensory condition. However, this comparison is invalid if different temporal windows are used on unisensory versus multisensory trials, or if the decision process differs in some other way across conditions (e.g. termination criteria; see below). Thus, a more general conception of optimality requires consideration of how the decision process unfolds in time.

This is just one example of a broader problem with the conventional approach to defining and testing for optimality: experimental conditions where only one cue is presented may not accurately quantify the reliability of the cues when presented together ([39,40], and see Zaidel & Salomon [41], for a more nuanced and expansive view on this topic). Nevertheless, one can at least investigate the time course of multisensory decisions by measuring RT (see below), and by relating stimulus fluctuations [37,42] or neural activity [43,44] to behaviour in a temporally resolved fashion. In addition to providing an update to the concept of optimality [45], studying the temporal properties of individual decisions—including how the brain decides *when* to decide—is essential if we wish to understand how they are strung together into sequences, as is the case during real-world navigation.

## 3. Two key ingredients for sequential decisions in complex environments

### (a) Decision termination and the speed-accuracy trade-off

The time it takes to reach a decision (perceptual, mnemonic or otherwise) has been a bedrock of quantitative psychology for many decades [46–49]. The need to place limits on the time of decision formation is especially salient during self-motion, where timely action can mean the difference between obstacle avoidance and collision, or catching versus missing a prey item. Yet we do not know exactly what determines the time of commitment to a heading judgement, a process made more complicated by the multiple, dynamic sources of information that must be taken into account.

A reasonable starting point is to assume, as is standard in the field, that the brain accumulates noisy evidence until an internal bound is reached, at which point the decision process is terminated and a choice is rendered [50]. Drugowitsch and colleagues therefore developed a multisensory drift-diffusion model in their investigation of choices and RT during multisensory heading discrimination [45]. As mentioned above, considering the dimension of time motivates an update to conventional theories of Bayes-optimal cue combination. In the classic (static) model [14,16], stimuli give rise to individual noisy estimates, and the multisensory estimate is their reliability-weighted average. By contrast, a dynamic model seeks to explain the combination of signals both across modalities and across time. In the model of Drugowitsch *et al*. [45], ‘momentary evidence’ at each time step is constructed by weighting visual and vestibular signals by their instantaneous reliability—which is a function of (i) a global stimulus property that varies across trials (e.g. visual motion coherence), and (ii) within-trial stimulus dynamics, namely its velocity or acceleration profile, respectively. The temporal accumulation of this evidence to a bound gives rise to both the choice and RT, and the height of the bound dictates the trade-off between speed and accuracy.

Crucially, when considered as a bounded process, multisensory decisions are not always more precise than unisensory ones, as predicted by the static model—but in that case they ought to be faster, and this is what Drugowitsch *et al*. [45] found. Thus, when optimality is defined at the level of the momentary evidence, discrimination thresholds alone are not sufficient to assess optimality, as increasing decision speed at the cost of accuracy can be optimal in terms of maximizing reward rate [51]. Another important contribution of this work is to derive a normative solution for decisions where evidence reliability varies over short time scales [52], a common feature of natural environments but one that is largely unaddressed in classic studies of perceptual decision-making (but see [53] for a thorough and timely review of more recent efforts)). It can be shown that optimal integration of time-varying evidence is theoretically tractable under certain assumptions [52], but whether and how the brain achieves this remains unresolved.

One approach to the question of neural implementation was undertaken by Hou *et al*. [32]. They suggested that integration of multiple dynamic evidence streams could be mediated by invariant linear combinations of neural inputs across time and sensory modalities, and observed neural activity consistent with this integration in the lateral intraparietal area (LIP) [32]. However, this experiment did not measure behavioural RT, and trial-averaged neural responses by themselves may not be diagnostic of bounded evidence accumulation [54]. Indeed, there is reason to wonder whether the brain actually uses a strategy of accumulating noisy samples of evidence in the heading task. The main motivation to accumulate evidence in the first place is to average out the noise, but this works best when the samples are independent [55] or at most weakly correlated. The degree to which self-motion fits the bill is unclear, given that the relevant signals have a high degree of autocorrelation (being tied to inertial motion, not an arbitrary pattern of inputs), and unique noise properties [56–58] whose implications for decision-making have not been fully explored.

Thus far the available evidence [32,45] (and see following section) is consistent with bounded accumulation, but it is still worth considering alternatives, such as taking a single ‘snapshot’ of evidence [38] at the predicted time of maximum information content (i.e. the peak of the velocity or acceleration profile [18]). At the other extreme, one might envision a continuous process best explained using elements of control theory [59,60], although the relationship between evidence and decision termination is less clear in such a framework. Previous work has shown that adjudicating candidate decision processes may require testing the same subjects in different task variants, for instance experimenter-controlled duration versus reaction-time versions [38]. Data from either variant by itself may be consistent with several distinct processes, so a more stringent test is to fit the data from one variant and use a subset of the fitted parameters to predict the other variant [38]. Neurophysiology could help as well, especially high-density recordings which permit single-trial analyses of decision variable representations [61–63]. This three-pronged strategy—behavioural readout of decision dynamics/termination, computational frameworks that accommodate time-varying signals, and neurophysiological approaches capable of linking the two—seems like a promising path towards expanding the neurobiology of decision-making to more natural multisensory contexts.

### (b) Confidence in multisensory decisions: candidate models and a pilot experiment

In visual perception, the framework of bounded evidence accumulation has been extended to explain a third key outcome inherent to the decision-making process: confidence, defined as the graded, subjective belief that the current or pending decision is correct. Considered an elemental component of metacognition, confidence is of long-standing interest to psychologists and philosophers of mind, and (alongside RT) has figured prominently in psychophysical theory and experiment for over a century [64–67]. Yet it also serves a practical purpose in natural behaviour. Most real-world decisions are not made in isolation but are part of a sequence or hierarchy in which the appropriate choice depends on the unknown outcome of earlier decisions. In such a scenario, confidence functions as a proxy for feedback, a prediction of accuracy that informs the next choice in a sequence—or more generally, adjustments of decision strategy [68,69] or learning rate [1,70].

Almost all models of confidence devote a key role to the strength of the evidence informing the accompanying choice. In models based on signal detection theory (SDT) [71,72], observations further away from a decision criterion (i.e. stronger evidence) are more likely to have arisen from one distribution over another, justifying higher confidence. When decision time is factored in and controlled by the subject, the accumulated evidence and the time taken to reach a decision maps onto the probability of making a correct choice, and this mapping (or an approximation thereof) could be learned and used to assign a degree of confidence [73,74].

Interestingly, these two frameworks offer distinct interpretations for the empirical relationship between confidence and stimulus strength [74,75]: confidence increases with stimulus strength on correct trials, but often (though not always) *decreases* with stimulus strength on error trials. SDT attributes this to the fact that an observation leading to an error must be closer to the criterion when *d*’ is large, whereas in accumulator models it can be explained by continued accumulation of contradictory evidence after an initial choice is made [74,76]. This discrepancy further underscores the relevance of time as a factor in decision-making, and especially confidence; indeed, failing to consider the time dimension can lead to misinterpretation of common measures of metacognitive performance [77].

The study of confidence in perception has typically considered situations in which the relevant sensory evidence arises from just one external source. However, most real-world sensory experiences consist of concurrent inputs from multiple modalities, and some basic unanswered questions arise when studying metacognition in a multisensory context [78]. For example, we do not know whether confidence is generated by the same cue-integration process (be it evidence accumulation, Bayesian inference or something else) underlying the decision itself. Alternatively, confidence could be computed by a parallel process that is not contingent on moment-by-moment sensory evidence, or by a post-decision accumulation process [79] with distinct termination criteria [80]. A quantitative link between choice and confidence was supported by perturbations of visual cortical neurons supplying the evidence in a random-dot motion task [81,82], but these studies did not rule out post-decisional processing, and the decision was based on a single modality.

To begin to address this question for multisensory decisions, we reasoned that behavioural measures of choice and confidence should demonstrate similar dependence on relative cue reliability, which is classically assessed using a cue-conflict manipulation [13,16,83]. When cues are placed in conflict, the resulting bias in the psychometric function reflects the weight assigned to each cue during discrimination. By randomly interleaving different levels of relative reliability, one can test whether cues are reweighted on a trial-by-trial basis, as predicted by normative theory and demonstrated empirically in previous work. What remains to be seen is whether confidence judgements reflect the same reliability-based cue weighting that has been observed in choices.

**Figure 2.**
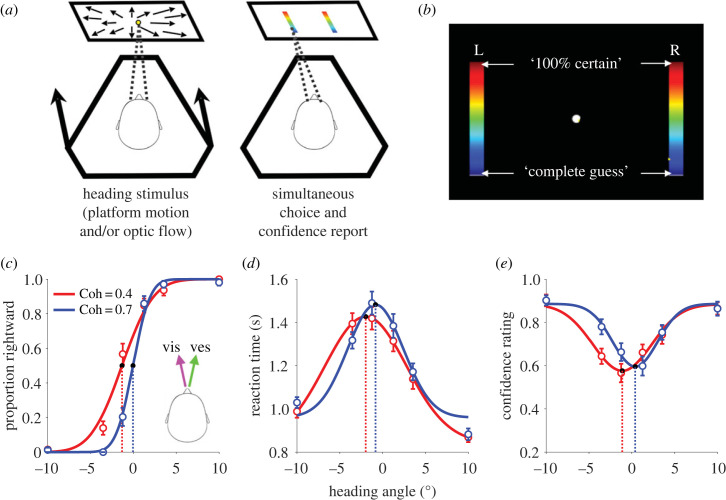
Pilot experiment with concurrent measurement of choice, RT, and confidence in heading discrimination. Human participants (*n* = 5 subjects, 4092 total trials) were seated in a cockpit-style chair within a motion platform, facing a rear-projection screen and eye-tracking camera. (*a*) Each trial consisted of a translational motion stimulus delivered visually (optic flow of a random-dot cloud with variable motion coherence, (Coh)) and/or by inertial motion of the platform. When ready, participants indicated whether their perceived heading was to the right or left relative to straight ahead, by making a saccade to one of two vertical colour-gradient bars. (*b*) Subjects were instructed to aim their saccade to a vertical position along the chosen target based on their confidence in the right/left choice, defined on a continuous scale from ‘complete guess’ to ‘100% certain’. (*c*) Proportion of rightward choices as a function of heading angle (positive = rightward) for cue-conflict trials where the visually-defined heading was displaced 1.5° to the left, and vestibular heading 1.5° to the right of the assigned heading angle on each trial. The two curves are for low (0.4, red) and high (0.7, blue) visual coherence. The results replicate the known effects of cue reliability on sensitivity (slope) and cue weights (bias) [11]. (*d*) Reaction time as a function of heading angle for the same conditions as in (*c*). (*e*) The same as (*d*) but for confidence ratings (mean saccade endpoint). Smooth curves are descriptive Gaussian fits (cumulative, regular or inverted), and dotted lines highlight the mean of the fitted Gaussians.

We adapted a well-established heading discrimination task [13] to include simultaneous reports of choice and confidence via a continuous rating scale [74]. The task also measures RT, allowing us to test whether a bounded accumulation process underlies all three behavioural variables in this task, as has been suggested for visual motion discrimination [75]. Human participants (*n* = 5) seated in a motion platform were instructed to report their heading (left or right) relative to a fixed reference of straight ahead. Each trial consisted of a translational heading stimulus comprising inertial motion and/or synchronous optic flow of a random-dot cloud. Participants indicated their choice and confidence by making a saccade to one of two colour-gradient bars (figure 2*a,b*). The top of the bar represented 100% certainty, while the bottom of the bars represented a complete guess. Participants were not given immediate feedback about their performance, but instead were shown their percentage of correct choices at the end of each set of 30 trials. Interleaved throughout the session were cue-conflict trials in which the heading angles specified by visual and vestibular cues were separated by ±3°, a subtle difference not readily noticeable and with no qualitative impact on overall confidence level. Relative cue reliability was controlled by the coherence of the random dots, also randomly interleaved.

Cue reliability was reflected in the relative slope of psychometric functions (figure 2*c*; compare low (red) versus high (blue) visual coherence)—and correspondingly in the relative width of the RT and confidence functions (figure 2*d*,*e*). At the same time, the psychometric functions reveal a bias indicative of trial-by-trial reliability-based cue weighting, as shown previously [13]. In the condition shown in figure 2*c*–*e*, vestibular heading was offset 1.5° to the right and visual 1.5° to the left of the angle specified by the abscissa, and hence participants made more rightward choices when the vestibular stimulus was more reliable (low visual coherence, red curve shifted to the left). Strikingly, the RT and confidence curves show very similar shifts (figure 2*d*,*e*), suggesting that the multisensory evidence guiding choice also underlies a degree of confidence in the choice [81]. Although it awaits quantitative confirmation in a larger dataset, this to our knowledge is the first indication that reliability-based cue combination at the level of choices also manifests in confidence and RT.

**Figure 3.**
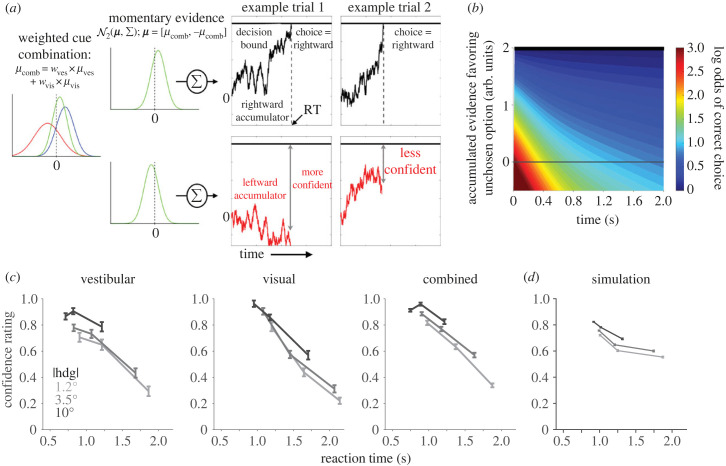
Multisensory bounded evidence accumulation model for unifying choice, reaction time (RT), and confidence. (*a*) Noisy momentary evidence is constructed as a reliability-weighted sum of heading evidence from visual and vestibular cues (red and blue distributions in leftmost panel). In this example, the two cues are conflicting and of different reliability (variance), and the resulting combined distribution (green curve) is biased towards the more reliable cue (here, shifted rightwards). Evidence favouring each of the two alternatives is accumulated separately and the choice is determined by which accumulator reaches its bound first; RT is given by the time of bound crossing (plus a small non-decision time). Two simulated trials are depicted. In both, the rightward accumulator wins the race, but in example trial 2, the decision maker is less confident because the losing accumulator was closer to the bound as compared to trial 1. (*b*) The model asserts that the brain has implicit knowledge of the relationship between the amount of evidence gathered by the losing accumulator and the probability (shown as the log odds) of making a correct choice, and uses this knowledge to compute confidence. (*c*) Confidence rating (mean ± s.e.) as a function of RT quantile, plotted separately for the three different heading angle magnitudes (|**hdg**|, leftward and rightward pooled) and three modality conditions: vestibular (platform motion only), visual (optic flow only), and combined. Data are from the same dataset as figure 2 (*n* = 5 subjects). (*d*) Simulations from the model also exhibit a negative relationship between confidence and RT, conditioned on |**hdg**|.

These preliminary findings are consistent with the hypothesis that confidence arises from the same evidence accumulation process that governs the decision (and termination thereof), rather than by a downstream or parallel mechanism independent of the reliability-based weighting process. We developed a multisensory decision model (figure 3*a*,*b*) that combines the reliability-weighted combination of momentary evidence from Drugowitsch and colleagues [45] with a two-dimensional accumulation process (anticorrelated race) that has successfully explained choice, RT, and confidence in a visual task [74,84]. In this model, evidence at each time step is drawn from a bivariate normal distribution with mean = [*μ*_comb_, −*μ*_comb_] and $\mathrm{covariance}=\left[\begin{array}{cc} 1 & \rho \\ \rho & 1 \end{array}\right]$, where the two dimensions correspond to the two choice alternatives (right versus left) and the evidence for each is partially anticorrelated (i.e. *ρ* < −0.5 [85]). The mean *μ*_comb_ is assumed to reflect the optimal weighting of evidence [45] and is therefore biased towards the more reliable cue (figure 3*a*, left). Evidence samples are fed into competing accumulators, and the winning accumulator determines both the choice and the decision time. Because the winning accumulator is always at a fixed value at decision time (i.e. the bound), confidence is determined by the status of the losing accumulator (figure 3*a*, right): the closer the losing race was to its bound, the lower the degree of confidence. This intuitive relationship is formalized by calculating the log odds of a correct choice as a function of accumulated evidence and time ([74,84]; figure 3*b*). The accumulation process jointly dictates choice, RT, and confidence—and because this process is downstream of the weighted cue-combination step, all three behavioural variables should exhibit the same reliability-based shift. This is what we observed in the pilot experiment (figure 2*c–e*).

Under common assumptions, the mathematics of drift-diffusion guarantees that the mapping between evidence and confidence is time-dependent [73,74]: a given level of evidence favouring the unchosen option maps onto a probability of being correct, but this probability decreases with elapsed decision time (figure 3*b*). The prediction is that confidence should be inversely related to decision time, even for a fixed level of stimulus strength [74] (here, heading angle; figure 3*d*). Our preliminary results are consistent with this prediction as well (figure 3*c*), which supports the notion that elapsed decision time is a vital part of the computation of confidence.

In summary, although more work is required to develop plausible alternatives for model comparison, the data are qualitatively consistent with a multisensory accumulator model such as the one in figure 3*a*. One major open question is where and how the weighted combination of momentary evidence is achieved in the brain. The output of this process—reliability-weighted *accumulated* evidence—appears to be represented in parietal and frontal cortices [32,86], but the upstream circuitry is unknown. As alluded to above, the dynamics of these accumulation-like signals differ for visual versus vestibular stimuli, suggesting they might originate from separate unimodal representations rather than a single multisensory representation such as in MSTd. Simultaneous recordings from (and perturbations of) multiple nodes in the self-motion network, along with downstream decision-related areas, may be needed to resolve this question.

### (c) Sequential self-motion judgements as a scaffold for navigation

Decision termination and confidence are critical in furnishing a decision-maker with the ability to make sequential judgements at appropriate intervals to achieve their goals. If external feedback about decision accuracy is available, decision-makers generally exhibit slower RT after errors, consistent with an evolving trade-off between speed and accuracy based on recent experience [68,87]. On the other hand, in the absence of external feedback, as is frequently the case for real-world decisions, the internal ‘feedback’ furnished by a representation of confidence could drive adjustments of decision policy, through a modification of the termination bound or accumulation process [88,89].

In the case of self-motion, an individual decision could equate to a prediction of body position or orientation in the near future, derived from an integration of multisensory evidence and expected dynamics (spatio-temporal priors) over some period of time [90]. Performed repeatedly, these perceptual decisions constitute the building blocks for a path integration process, with the decision at one time-step feeding into the next. The perception of egocentric heading for path integration is a key component of real-world navigation, although successful navigation also draws on salient environmental information [91], and a psychological sense of self-location [92].

Since path integration accumulates errors, monitoring one's ongoing certainty and incorporating this into an evolving behavioural strategy could be quite useful, particularly when knowledge of one's current position or goal location is incomplete. This idea goes beyond using uncertainty to update a position estimate, in the manner of a Kalman filter [93,94]. Our conjecture is that a metacognitive certainty judgement accompanies each position update and can be used to guide higher-order decisions, for instance whether to maintain or reverse the current course, or to stop and sample more information.

During truly continuous natural behaviour, one might assume that the time interval between successive position estimates should reach zero at its limit. However, given that upcoming spatio-temporal sequences of natural motion stimuli are generally predictable to the agent (through motor efference and plentiful experience), the interval between updates could be adjusted based on inferred changes in control dynamics of the current environment. In other words, although continuous computation of self-motion is necessary for reflexes and postural control, it may be unnecessary to maintain a *perceptual* estimate of self-motion at all times, agnostic to the current behavioural state. Instead, the brain could reduce the computational burden by only consulting the self-motion system when a salient transition or event boundary is detected. This might correspond to a change in expected reliability of different sensory cues, or in the control dynamics needed for that part of the environment [90], such as a change in ambient light level or terrain. The relationship between inputs (i.e. motor commands), and outputs (body motion) is predictable from an internal model of control dynamics and the autocorrelation structure of self-motion, but in situations where the environment changes rapidly, confidence judgements associated with each position update could play a key role. In the next section, we discuss increasing efforts to develop more closed-loop behavioural tasks to address the computational and neural bases of self-motion perception along these lines.

## 4. Where are we heading? Naturalistic self-motion

The motivation for framing self-motion perception as a decision-making process arises from a consideration of the goal it ultimately serves, which is to allow an agent to accurately orient and navigate within its environment. In such dynamic environments, the brain must use perceptual observations to guide subsequent actions, and, as described by classical reinforcement learning models, actions are chosen to maximize the likelihood of immediate and/or future desirable outcomes [95]. An agent may also select actions, including eye movements, to improve its ‘vantage point’ for new observations that could lead to desirable outcomes further down the line [96]. Previous studies of self-motion perception, by operating under fixed-gaze conditions and soliciting single, binary responses to passively experienced stimuli, decouple this natural link between perception and action, which may impose fundamental limitations on our ability to understand general principles of neural computation and behaviour.

**Figure 4.**
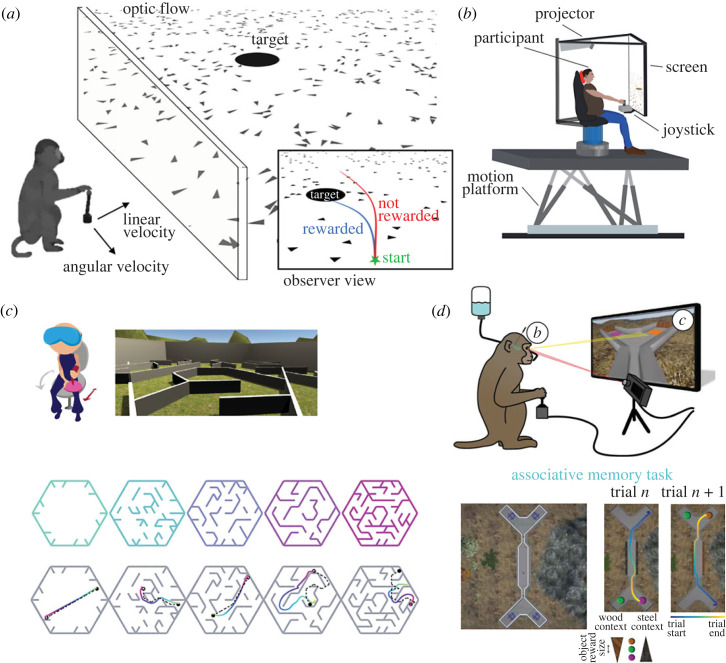
Virtual reality (VR) paradigms for bridging self-motion perception and spatial navigation. (*a*) Schematic of ‘fireflies’ task [88–92]. Human or monkey subjects control linear and angular velocity in a virtual environment via a joystick to navigate to a briefly shown target, using ground-plane optic flow cues. Reward is obtained by stopping within a zone surrounding the location of the target (inset). Reproduced with permission from [102]. (*b*) Experimental set-up in a multisensory fireflies task. Subjects sit within a 6-degree-of-freedom motion platform with coupled rotator. Reprinted from Stavropoulos *et al*. [90], Creative Commons Attribution License. (*c*) (Top) VR navigation task in [103]. Human subjects wearing a VR headset navigated in a virtual maze, using a joystick to control fore-aft motion and turning by rotating in a swivel chair. Screenshot shows first-person view. (Bottom) Layout of maze arenas used in the task, with increasing complexity defined by obstacle configuration. Example subject trajectories are shown in each maze. Empty and filled circles denote start and end point, respectively. Black dashed line shows optimal trajectory. Reprinted from Zhu *et al*. [103], Creative Commons Attribution License. (*d*) Other applications of monkey VR include tests of associative learning and memory, with concurrent recording in the hippocampus and prefrontal cortex. Reproduced with permission from [104].

Approximating real-world decision-making about self-motion can still be achieved within the laboratory and build on existing understanding of perception, by using natural planning strategies in animal behaviour, and tracking continuous variables over individual trials to generate rich time-varying behavioural data. The appeal to naturalistic, evolutionarily ingrained, behaviours in such tasks provides an opportunity to explore decision-making processes during self-motion across species, often while avoiding the need for heavily over-trained animals. Adopting this approach, a recent set of experiments instructed human and non-human primate subjects to navigate with a joystick to memorized target locations (fireflies) in a virtual environment with ground-plane optic flow cues [97–101] (figure 4*a*, and see [105]). In this task, the evolution of the optic flow pattern is driven by the subject's own movements in the virtual environment, maintaining a link between perception and action. The task also emulates foraging behaviour, a natural example of the use of path integration. Eye movements over the course of each trial, lasting up to several seconds, reliably track the evolving latent location of the target and correlate with success, reflecting the subject's dynamic belief about the target location [98]. Perturbations in optic flow or joystick gain indicate that humans and monkeys rely on optic flow to perform this task [101], and subjects are also able to rapidly and effectively generalize to novel task variants, including joystick gain changes, moving latent targets, selecting between two targets, and chasing an inexhaustible supply of flashing ‘fireflies’ over tens of minutes [102].

A multisensory version of the firefly paradigm used gradual across-trial fluctuations in joystick parameters to assess the contribution of visual and vestibular cues and control dynamics to path integration [90]. To overcome limitations in the linear trajectories possible with the motion platform (figure 4*b*), Stavropolous and colleagues made use of the tilt-translation (gravito-inertial) ambiguity to provide combined rotation and translation of the platform (and accompanying optic flow stimulus in combined visuo-vestibular trials), which would be perceived equivalently to the linear accelerations intended by subjects' joystick commands. Successful navigation in this task depends not only on integration of momentary sensory evidence, but on combining this information with an accurate internal model of latent control dynamics [90]. In particular, vestibular inputs alone generally provided unreliable cues for navigation compared to optic flow, and better performance was mainly seen in the presence of sustained acceleration, consistent with the dominance of acceleration-dependent responses within the vestibular processing hierarchy [106,107]. This also complements the observation that longer duration stimuli elicit greater reliance on visual (velocity) information [108], although velocity estimation from optic flow can still result in systematic undershoot biases owing to a prior expectation of slower velocities [97,99].

Joysticks, and the ‘continuous’ behaviour they permit, thus provide a useful tool to re-establish the link between perception and action, a link broken in classic psychophysical tasks with independent stimuli and discrete end-of-trial responses. Yet, there remains an abstraction from true self-generated motion, or at least a distinct mapping between intended actions and the gamut of idiothetic cues of self-motion (i.e. vestibular, proprioceptive and motor efference). This distinction has meaningful consequences for the central processing of vestibular information [109]. While steering-based navigation with real vestibular cues elicits responses in the brainstem vestibular nuclei, these responses are attenuated during true active self-motion [110], probably because the reafferent signals from actively generated movements are cancelled through a comparison with expected consequences within the cerebellum [111]. Nevertheless, modelling work [112] demonstrates that both active and passive vestibular stimuli can be processed within the same internal model which makes continuous predictions of expected sensory feedback. This validates the use of externally applied motion stimuli in experimental settings, and their relevance in real-world self-motion, such as during perturbations, or mismatches between planned and executed movements [112].

### (a) Active motion and virtual reality in monkeys: new applications

Closed-loop tasks which retain some grounding in established theory and produce rich behaviour in primate subjects open exciting new avenues for probing the neural basis of self-motion perception. Simultaneous recordings in MSTd and the dorsolateral prefrontal cortex in the fireflies task suggest that these areas, and the coupling between them, may represent important latent variables such as angular distance to the target [100], consistent with task strategies inferred from gaze location [98]. Going forward, it will be important to reconcile MSTd responses during goal-directed navigation with existing foundations from classic paradigms. This will help to understand the circumstances in which MSTd responses may shift from encoding current heading during smooth pursuit eye movements [113] to encoding angular or linear distance to a goal location [100,114]. It may also be interesting to ask whether variability in eye and joystick position could be a useful proxy measure of confidence, assuming that a greater degree of confidence is associated with greater movement vigor [115,116] or fewer changes-of-mind [84].

Gaze patterns have been shown to form an integral component of planning behaviour in larger and yet more complex virtual environments. Zhu *et al*. [103] found that human subjects' eye movements map out future trajectories to the goal, and relevant subgoals (turning locations), in an arena navigation task, emphasizing the value of natural oculomotor behaviour in untangling deliberations and strategies during sequential decision-making. In this task, fore-aft motion was controlled via a joystick, but subjects could rotate in the VR environment through actual movement on a 360° swivel chair. Although the primacy of actively sampled visual input is clear, such set-ups could provide cross-modal inputs from vestibular and proprioceptive systems of the head and neck, offering opportunities to extend investigations of multisensory self-motion perception into the realm of flexible goal-directed navigation. This less-constrained style of VR experiment has found its way into several domains of non-human primate systems neuroscience, including studies focused on learning and memory [104] (figure 4*d*), abstract decision-making [117] and visual experience [118]—further greying the traditional boundaries between these areas of study.

The emerging trend for more complex, naturalistic tasks, and the ability to extract meaningful, quantitative insights from them, is symbiotic with increasingly available technologies for large-scale recording of neural populations [119] and sophisticated analytical tools [120–122]. Such recordings have already highlighted how perception and behaviour arise from coordinated activity patterns across multiple areas [123–126], implementing computations that evolve over time and are best understood at the population level [127–133]. Making sense of high-dimensional neural data in the context of self-motion may require not only analysing the dynamics of neural activity on single trials [121,134] but also relating these dynamics to suitably high-dimensional behaviour, for instance by using new methods for pose estimation in unrestrained animals [135–138].

## 5. Summary and outlook

Self-motion perception is a fundamentally multimodal cognitive process, essential for survival in mobile organisms. Particularly indispensable to this process are inertial motion signals arising from the peripheral vestibular apparatus—semicircular canals and otolith organs—and optic flow responses to global motion of the visual scene. Classical psychophysical studies in humans and non-human primates, often combined with neurophysiology and normative theoretical approaches, have exposed key principles of heading perception grounded in the idea that sensory information is inherently probabilistic. At the cortical level, multisensory heading perception probably involves the concerted interaction of multiple areas, and ongoing work continues to piece together the response properties of cells across these areas, their potential roles, and the interactions between them.

The introduction of RT measurements in multisensory heading discrimination is an important step towards understanding self-motion decisions in an ecological setting and clarifying what is meant by ‘optimality.’ At both the computational and neural levels, subjective reports of certainty during multisensory heading perception could also uncover important features of time-dependent decision-making and sequential judgements of heading.

Nonetheless, these additions remain grounded in the passive, independent-trial approach of conventional psychophysics and neurophysiology, which is bound to provide a limited view of computation during natural self-motion and its associated decisions. There remain significant open questions about how and where noisy sensory information is integrated across modalities, over time, and combined with internal models of dynamics for the perception of self-motion in complex environments. The ability to uncover latent variables from continuous behaviour and neural population activity will be essential to this endeavour, unveiling mechanisms that bridge time scales from individual decisions to goal-directed navigation.

## Data Availability

Data are freely available from the GitHub repository: https://github.com/Fetschlab/dots3DMP_humanPilotData [139].
